# 2782. In vitro activity of ceftazidime-avibactam against Gram-negative strains in patients with bacteremia and skin and soft-tissue infections in Colombia 2019-2021

**DOI:** 10.1093/ofid/ofad500.2393

**Published:** 2023-11-27

**Authors:** Elkin Lemos-Luengas, Sixta Romelia Rentería-Valoyes, Diana Marcela Almario Muñoz, Carlos David Gamboa Orozco, Juan Camilo Olivella Gómez, Jorge Ramos-Castaneda

**Affiliations:** Innovative Medicines Business, Pfizer Colombia, Bogotá, Distrito Capital de Bogota, Colombia; Pfizer Colombia, Bogota, Distrito Capital de Bogota, Colombia; Innovative Medicines Business, Pfizer Colombia, Bogotá, Distrito Capital de Bogota, Colombia; Innovative Medicines Business, Pfizer Colombia, Bogotá, Distrito Capital de Bogota, Colombia; Innovative Medicines Business, Pfizer Colombia, Bogotá, Distrito Capital de Bogota, Colombia; Research Group Innovación y Cuidado, Faculty of Nursing, Universidad Antonio Nariño, Neiva, Huila, Colombia

## Abstract

**Background:**

In vitro activity of Ceftazidime-avibactam (CAZ-AVI) in patients with bacteremia and skin and soft tissue infections (SSTIs) is unknown. The study aimed to analyze the in vitro antimicrobial activity of CAZ-AVI and other antimicrobials against Gram-negative bacilli collected in hospitals in Colombia in patients with bacteremia and SSTIs.

**Methods:**

Enterobacterales and *P. aeruginosa* from patients with bacteremia and SSTIs were analyzed. Strains were collected from four ATLAS-reporting hospitals in Colombia between 2019 and 2021.

**Results:**

Enterobacterales:

A total of 600 Enterobacterales were collected. The most susceptible antimicrobials to Enterobacterales were CAZ-AVI (96.5%), tigecycline (95%), and amikacin (92.17%). The incidence of carbapenem-resistant Enterobacterales (CRE) was 13.5% (n= 81), and the antimicrobials with the best activity were tigecycline (93.83%), CAZ-AVI (74.07%), and amikacin (50.62%) (Figure 1).

Tigecycline, CAZ-AVI, and amikacin were the antimicrobials with the best in vitro activity against MDR Enterobacterales, extended-spectrum β-lactamase-producing (ESBL) Enterobacterales, and KPC-producing Enterobacterales (Figure 2).

*K. pneumoniae* (n= 198), *E. coli* (n= 167), *Enterobacter cloacae* (n= 46), and *Serratia marcescens* (n= 41) were the strains most frequently isolated (Table 1). Tigecycline and CAZ-AVI had excellent in vitro activity against all four species, as well as for those ESBL-producers, MDR, and carbapenem-resistant (CR).

*P. aerugniosa:*

A total of 259 *P. aeruginosa* were analyzed. CAZ-AVI was the antimicrobial with the best in vitro activity against *P. aeruginosa* with a susceptibility of 83.4%, as well as against CR (S= 44%), MDR (S= 50.58%), and DTR *Pseudomonas* (S= 33.33%).

Figure 1 Antimicrobial activity among isolates of Enterobacterales and Carbapenem-resistant Enterobacterales (CRE) collected in Colombia between 2019 - 2021

**Figure:**
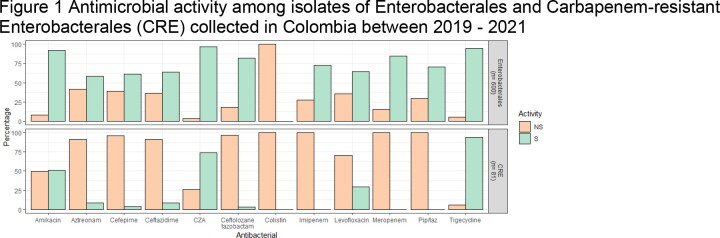

S: susceptible; NS: Not susceptible; Pip/taz: Piperacillin-tazobactam; CZA: Ceftazidime-Avibactam; S: susceptible; NS: Not susceptible; CRE: Carbapenem-resistant Enterobacterales

Figura 2 Not susceptibility among isolates of Extended-spectrum β-lactamase Enterobacterales, Multidrug-resistant Enterobacterales, Klebsiella pneumoniae carbapenemase (KPC)-producing enterobacterales, and metallo β-lactamase producing enterobacterales in Colombia

**Figure:**
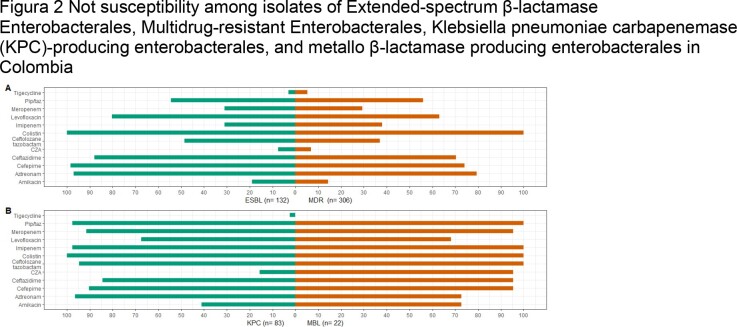

A. Not susceptibility among isolates of Extended-spectrum β-lactamase enterobacterales (green color) and Multidrug-resistant Enterobacterales (orange color) B. Not susceptibility among Klebsiella pneumoniae carbapenemase (KPC)-producing enterobacterales (green color) and metallo β-lactamase producing enterobacterales (orange color) Pip/taz: Piperacillin-tazobactam; CZA: Ceftazidime-Avibactam; MDR: Multidrug-resistant

**Figure:**
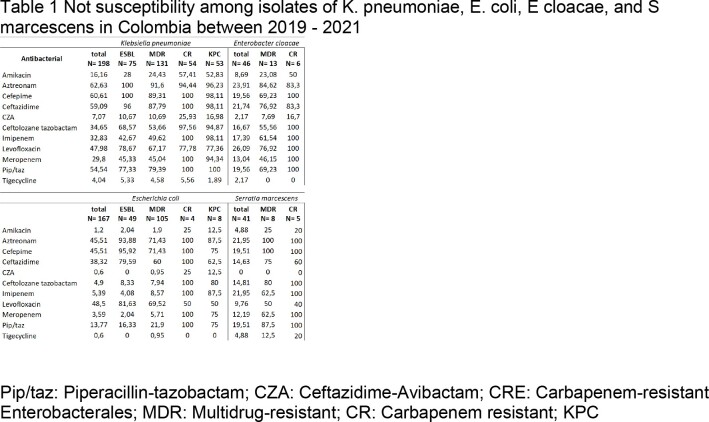

**Conclusion:**

Tigecycline and CAZ-AVI were the two antimicrobials with excellent in vitro activity against clinical specimens from patients with bacteremia and SSTIs caused by CRE, MDR Enterobacterales, and ESBL- and KPC-producing Enterobacterales. Against MDR, CR, and DTR *P. aeruginosa*, CAZ-AVI and amikacin were the antimicrobials with the best in vitro activity (Figure 3).

**Figure:**
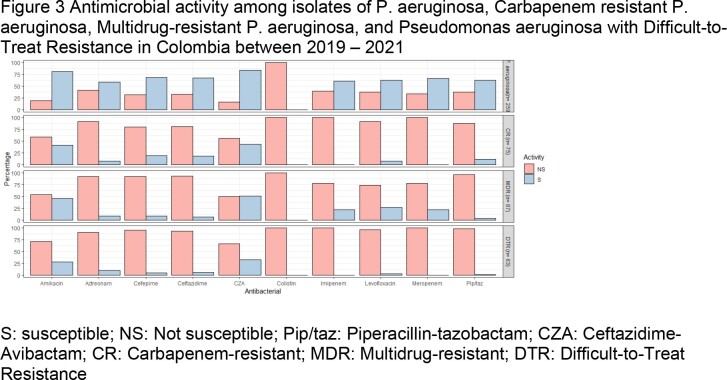

**Disclosures:**

**Elkin Lemos-Luengas, MD MSc PhD**, Pfizer Colombia: Honoraria **Sixta Romelia Rentería-Valoyes, Md**, Pfizer: Senior Associate Medicines Business /Medical Scientific Liaison MD for Pfizer Colombia **Diana Marcela Almario Muñoz, MD, MSc**, Pfizer Colombia: Medical Scientific Liaison **Carlos David Gamboa Orozco, Md**, Pfizer: Medical Scientific Liaison for Pfizer Colombia **Juan Camilo Olivella Gómez, n/a**, Pfizer Colombia: Medical Student **Jorge Ramos-Castaneda, PhD**, Pfizer: Advisor/Consultant

